# Severity of diabetic foot ulcers at various levels of HbA1c

**DOI:** 10.11604/pamj.2026.53.4.50436

**Published:** 2026-01-05

**Authors:** Lakhveer Rathi, Santosh Kumar, Rafique Hussain, Adnan Abdalla, Shahnawaz, Musab Suliman, Hina Ismail

**Affiliations:** 1 Department of Medicine, Liaquat University of Medical and Health Sciences (LUMHS), Jamshoro, Pakistan,; 2 University Hospital Limerick, Limerick, Ireland,; 3 Sindh Institute of Urology and Transplantation (SIUT), Karachi, Pakistan

**Keywords:** Diabetic foot ulcer, HbA1c, glycemic control, Wagner classification, diabetes mellitus

## Abstract

Diabetic foot ulcer (DFU) is a major complication of diabetes mellitus and a leading cause of lower-limb amputation. Poor glycemic control has been implicated in DFU development and progression. This study explored the relationship between HbA1c levels and DFU severity using Wagner’s classification among patients with type 2 diabetes. In this cross-sectional study conducted over six months at Liaquat University of Medical and Health Sciences, Jamshoro, 64 patients aged 40-80 years with type 2 diabetes for >5 years and HbA1c >6.5% were enrolled. DFU severity was graded using Wagner’s classification (grades 1-5). Glycated hemoglobin (HbA1c) levels were categorized into four groups. Associations were assessed using chi-square tests. Mean age was 59.8 ± 8.7 years, with 60.9% males. Mean HbA1c was 8.9 ± 1.3%. Advanced ulcers (Wagner grades 4-5) were present in 51.5% of patients. Patients with HbA1c >8.5% accounted for 76.9% of those with grade 4-5 ulcers. HbA1c levels were significantly associated with DFU severity (χ^2^= 28.6, p = 0.002). Higher HbA1c levels were significantly associated with more severe DFUs. Optimizing long-term glycemic control may reduce ulcer severity and related complications.

## Introduction

Diabetic foot ulcer (DFU) represents one of the most severe complications of diabetes mellitus [1]. It arises due to multifactorial pathological processes, including peripheral neuropathy, peripheral arterial disease, and impaired wound healing [2]. Globally, 9.1-26 million individuals with diabetes experience DFU annually, with a lifetime prevalence ranging from 19-34% [3]. Diabetic foot ulcers are a leading cause of lower-limb amputation, resulting in prolonged hospital stays, increased mortality, and substantial healthcare costs [4].

Poor glycemic control plays a central role in DFU development and progression [5]. Hyperglycemia impairs leukocyte function, reduces tissue oxygenation, and disrupts collagen synthesis, delaying wound healing and increasing infection risk [6]. Glycated hemoglobin (HbA1c) reflects average plasma glucose over the preceding 8-12 weeks and is an established measure of long-term glycemic control [7]. Higher HbA1c levels correlate with greater DFU severity, prolonged healing, and increased amputation risk [8]. Wagner classification remains widely used to grade DFU severity, ranging from superficial ulcers (grade 1) to extensive gangrene (grade 5) [9]. Studies, such as Farooque *et al*. have demonstrated a statistical correlation between HbA1c and Wagner grades, with 59% of patients with grade 4-5 ulcers exhibiting HbA1c >8.5% [10]. However, literature reports are inconsistent, with confounding factors like diabetes duration, obesity, and vascular disease influencing outcomes. Local data from Pakistan on the association between HbA1c and DFU severity are lacking. This study aimed to assess the relationship between HbA1c levels and DFU severity in type 2 diabetes patients.

## Methods

**Study design and setting:** a hospital-based cross-sectional study was conducted at the Department of Medicine, Liaquat University of Medical and Health Sciences (LUMHS), Jamshoro, Pakistan, from 1^st^ January 2023 to 30^th^ June 2023.

**Participants:** patients aged 40-80 years with type 2 diabetes for >5 years and HbA1c >6.5%, diagnosed with DFU, were included. Exclusion criteria comprised type 1 diabetes, non-diabetic traumatic ulcers, prior amputations for non-diabetic causes, and a history of autoimmune, hematological, or malignant diseases.

**Sample size:** based on Wagner grade proportions reported by Farooque *et al*. [10], a sample size of 64 patients was calculated using PASS software to detect an effect size of 1.07 with 90% power and significance level 0.01. Consecutive sampling was used.

**Data collection:** after obtaining informed consent, eligible participants were identified through daily inpatient and outpatient medical ward admissions at LUMHS Jamshoro. All enrolled patients meeting the inclusion criteria during the study period were approached. After screening for eligibility, patients were informed about the study objectives and procedures. Written informed consent was obtained before enrollment. Glycated hemoglobin was measured at the hospital's central laboratory using a high-performance liquid chromatography (HPLC)-based assay, which is standardized to the National Glycohemoglobin Standardization Program (NGSP). Blood samples were obtained at the time of admission or clinic visit, and HbA1c values reflected average glycemic control over the preceding 8-12 weeks. A consecutive non-probability sampling technique was used to minimize selection bias. Ulcer severity was assessed clinically by the treating physician using Wagner's classification system, which grades diabetic foot ulcers as follows: grade 1: superficial ulcer without tendon or bone involvement; grade 2: deep ulcer penetrating to tendon or joint capsule; grade 3: deep ulcer with abscess, osteomyelitis, or joint sepsis; grade 4: localized gangrene of the forefoot or heel; grade 5: extensive gangrene involving the entire foot. For analytical purposes, ulcers were also grouped as mild (grades 1-2), moderate (grade 3), and severe (grades 4-5). Diabetic foot ulcers were graded using Wagner's classification (grades 1-5). HbA1c was categorized as 6.5-7.5%, 7.6-8.5%, 8.6-9.5%, and >9.5%.

**Statistical analysis:** data were analyzed using SPSS version 27. Continuous variables (age, duration of diabetes, HbA1c) were assessed for normality and summarized as mean ± standard deviation. Categorical variables (sex, HbA1c categories, Wagner grades) were presented as frequencies and percentages. The primary research question-the association between glycemic control and diabetic foot ulcer severity-was examined by cross-tabulating HbA1c categories with Wagner grades. The Chi-square test of independence was used to assess the relationship between categorical variables. When expected cell counts were <5, Fisher's exact test was applied. The measure of association was expressed using the chi-square statistic (χ^2^) with corresponding degrees of freedom and p-value. A two-tailed p-value <0.05 was considered statistically significant.

**Ethical considerations:** ethical approval was obtained from the Ethics Research Committee of LUMHS (approval no: ERC/LUMHS/2024/DFU-021). Written informed consent was obtained from all participants. Confidentiality and anonymity were strictly maintained.

## Results

A total of 64 patients with diabetic foot ulcers were included in the analysis. The mean age was 59.8 ± 8.7 years, and the majority were male (60.9%). The mean duration of diabetes was 12.3 ± 5.6 years, and the mean HbA1c level was 8.9 ± 1.3%. More than half of the patients (51.5%) presented with advanced ulcers (Wagner grades 4-5) (Table 1). A statistically significant association was observed between HbA1c levels and diabetic foot ulcer severity (χ^2^= 28.6, df = 12, p = 0.002). Patients with HbA1c levels greater than 8.5% constituted the majority of severe ulcers, accounting for 76.9% of Wagner grade 4-5 cases. In contrast, patients with HbA1c ≤8.5% were more likely to present with milder ulcer grades (Wagner grades 1-2) (Table 2).

**Table 1:** baseline characteristics of study population (n= 64)

Variable	Mean ± SD/n (%)
**Age (years)**	59.8 ± 8.7
**Gender**	
Male	39 (60.9)
Female	25 (39.1)
**Duration of diabetes (years)**	12.3 ± 5.6
HbA1c (%)	8.9 ± 1.3
**Wagner grade of DFU**	
Grade 1	4 (6.3)
Grade 2	6 (9.4)
Grade 3	21 (32.8)
Grade 4	23 (35.9)
Grade 5	10 (15.6)
**HbA1c categories**	
6.5 - 7.5%	11 (17.2)
7.6 - 8.5%	15 (23.4)
> 8.5%	38 (59.4)

DFU: diabetic foot ulcer, HbA1c: Glycated hemoglobin, SD: standard deviation

**Table 2:** association between HbA1c levels and Wagner grade of diabetic foot ulcer

HbA1c level (%)	Grade 1 (n)	Grade 2 (n)	Grade 3 (n)	Grade 4 (n)	Grade 5 (n)	Total (n)	P-value
6.5 - 7.5	2	3	4	2	0	11	0.002
7.6 - 8.5	1	2	7	3	2	15	
8.6 - 9.5	1	1	7	9	4	22	
> 9.5	0	0	3	9	4	16	
**Total**	4	6	21	23	10	64	

Chi-square = 28.6; df = 12; p = 0.002

## Discussion

The findings of this study highlight a significant correlation between elevated HbA1c levels and greater severity of DFUs. Patients with HbA1c >8.5% were more likely to present with advanced Wagner grade ulcers. These findings are consistent with Farooque *et al*. [10] and Akyüz *et al*. [8], highlighting that chronic hyperglycaemia impairs wound healing, promotes infection, and increases ulcer progression. Our research offers local insights from Pakistan, where delayed presentation and inadequate glycaemic control are prevalent. The high proportion of advanced ulcers reflects gaps in preventive foot care, patient education, and timely interventions. Early identification of patients with persistent hyperglycaemia may enable targeted preventive strategies, including multidisciplinary care and structured foot clinics. Limitations include the cross-sectional design, modest sample size, and absence of detailed vascular assessment. Future longitudinal studies could clarify whether tighter glycemic control improves ulcer outcomes.

## Conclusion

Higher HbA1c levels, particularly >8.5%, are significantly associated with more severe DFUs. Implementing strict glycemic control, early screening, patient education, and multidisciplinary care strategies is crucial in reducing the impact of advanced diabetic foot complications.

### What is known about this topic

- Poor glycemic control is a recognized risk factor for diabetic foot ulcers;
- Glycated hemoglobin reflects long-term glycemic status and correlates with diabetic complications;
- Wagner’s classification is widely used to grade DFU severity.

### What this study adds

- This study demonstrates a strong association between HbA1c >8.5% and advanced DFU grades;
- It provides local data from a tertiary care center in Pakistan;
- It also supports early risk stratification of DFU patients based on HbA1c levels.
